# Vagus nerve-fibre preservation during resection of vagal paraganglioma: a case series with patient-reported outcomes

**DOI:** 10.1093/jscr/rjag695

**Published:** 2026-09-11

**Authors:** Madeline Leadon, Theodore Howard, Alistair Ovenell, Peter M Clarke, Jonathan M Bernstein

**Affiliations:** Department of Otolaryngology–Head and Neck Surgery, Imperial College Healthcare NHS Trust, Charing Cross Hospital, London W6 8RF, United Kingdom; Department of Otolaryngology–Head and Neck Surgery, Imperial College Healthcare NHS Trust, Charing Cross Hospital, London W6 8RF, United Kingdom; Department of Otolaryngology–Head and Neck Surgery, Imperial College Healthcare NHS Trust, Charing Cross Hospital, London W6 8RF, United Kingdom; Department of Otolaryngology–Head and Neck Surgery, Imperial College Healthcare NHS Trust, Charing Cross Hospital, London W6 8RF, United Kingdom; Department of Otolaryngology–Head and Neck Surgery, Imperial College Healthcare NHS Trust, Charing Cross Hospital, London W6 8RF, United Kingdom; Department of Surgery and Cancer, Imperial College London, London, United Kingdom

**Keywords:** paraganglioma, vagus nerve, nerve sparing, quality of life, patient-reported outcome measures

## Abstract

Vagal paragangliomas are rare neuroendocrine tumours of the vagus nerve. Surgery typically causes vagus nerve paralysis with impairments of voice and swallow, and sometimes long-term gastrostomy dependence. We report a series of seven patients who underwent resections of enlarging, benign, non-functional vagal paragangliomas at a tertiary centre with attempted nerve-fibre preservation. MD Anderson Dysphagia Inventory (MDADI) and University of Washington Quality of Life scores were obtained by telephone interviews of six patients. Nerve-fibre preservation was achieved in three patients (43%), versus 5% reported in published systematic reviews. The preservation group had superior swallowing (mean MDADI composite 91 versus 66) and quality of life. No patient required tracheostomy or gastrostomy, and no tumour recurrence was identified at a mean follow-up of 10.5 years (range 6.4–12.7). Nerve-fibre preservation, when anatomically feasible, may reduce surgical morbidity without compromising local tumour control. Regionalisation of paraganglioma care and multi-centre data are needed.

## Introduction

Paragangliomas (PGL) are rare neuroendocrine neoplasms arising from neural crest derivatives. PGL of the head and neck (PGLHN) arise from parasympathetic glomus cells associated with the glossopharyngeal and vagus nerves, with an incidence of ~1:30 000 [1]. Vagal PGL arise from paraganglionic tissue along the vagus nerve and may present with a neck mass (70%), hoarseness (17%) or dysphagia (11%), or as an incidental finding at imaging (13%) [2]. A familial tendency is seen in ~40% of PGL (more than any other tumour affecting adults) which is frequently associated with pathogenic variants in succinate dehydrogenase (*SDH*) genes [1, 3]. Approximately 5% of PGLHN are metastatic and under 5% secrete catecholamines [4].

PGLHN should be managed at specialist centres owing to their rarity, complexity, and potential sequelae [1]. Treatment options include surveillance, surgery, and radiotherapy. Surveillance is appropriate for tumours that are benign, solitary, non-functional and asymptomatic [5]. In these circumstances, surgical resection may still be undertaken if the tumour is growing. Surgery involving transection of the vagus nerve on either side of the tumour causes vagus nerve paralysis and consequent impairments of articulation, voice, and swallow, with the potential for gastrostomy tube dependence long term [1, 6].

Vagus nerve-fibre sparing surgery is only reported rarely in the literature. In one retrospective study of 16 patients, nerve-fibre sparing was undertaken in four patients, with continued vocal fold motion and no aspiration occurring in two patients, while tumour recurrence occurred in one nerve-spared patient and two who had nerve resections [6]. A systematic review of 226 vagal PGL found the vagus nerve was functionally preserved in only 11 patients (5%), with tumour control in 98% of complete resections [7].

The present series reports a single-centre experience of vagal PGL resections with attempted nerve-fibre preservation, with patient-reported outcome measures (PROMs) comparing the preservation and transection groups.

## Case series

Seven patients with radiologically confirmed, enlarging vagal PGL who underwent surgical resections at a tertiary head and neck unit between January 2007 and November 2016 were identified retrospectively from operative records. The consensus-based clinical case reporting (CARE) guideline was followed. Mean follow-up was 10.5 years (range 6.4-12.7).

Examination included flexible nasopharyngolaryngoscopy, assessment of cranial nerve function, and auscultation for bruit [1]. All patients underwent contrast-magnetic resonance imaging (MRI) scans of the neck and some underwent whole-body MRI scanning to screen for multifocal disease, phaeochromocytoma, and distant metastases [1]. Metanephrines were tested by 24-hour urine collection or plasma at diagnosis [1]. All patients were offered genetic testing.

All patients were operated on by a single surgeon (PMC). Nerve-fibre preservation was attempted in all cases. Where vagus nerve fibres overlay the tumour capsule, and an identifiable plane existed between nerve fascicles and the capsule permitting sharp dissection without entering the tumour, those fibres were dissected and preserved. Six patients completed the MD Anderson Dysphagia Inventory (MDADI) [8] and University of Washington Quality of Life questionnaire (UW-QOL) patient-reported outcome measures (PROMs) by telephone interview in January 2023, at a mean of 10.1 years (range 6.4-12.3) after surgery; the seventh patient died from unrelated causes at 12.7 years follow-up, and before PROMs were collected for the present study.

Demographics and outcomes are summarised in Table 1. Mean age was 54 years (range 37–74). Three patients had multifocal PGL and three had pathogenic variants in *SDH*, consistent with the high familial predisposition in PGLHN. Pre-operative metanephrines were normal in all cases. One patient had embolisation pre-operatively. None had metastatic disease.

**Table 1:** Patient demographics and outcomes.

Characteristic	n (%) or mean [range]
Sex (male:female)	3 (43): 4 (57)
Age, years	54 [37–74]
Neck mass	4 (57)
Hoarseness	3 (43)
Tumour size, cm	3.8 [2.2–7.0]
Multifocal disease	4 (57)
*SDH* pathogenic variant confirmed	3 (43)
Pre-operative embolisation	1 (14)
Nerve-fibre preservation achieved	3 (43)
Post-operative radiotherapy	3 (43)
Vocal fold medialisation	5 (71)
Tracheostomy	0
Gastrostomy	0
Tumour recurrence	0
Horner's syndrome	4 (57)
Hypoglossal nerve paresis	3 (43)
Follow-up, months	107 [60–147]

Vagus nerve-fibre preservation was achieved in three patients (43%). Of these, two patients underwent subsequent vocal fold medialisations and one had successful laryngeal reinnervation. Three patients had complete vagus nerve transections, all of whom consequently underwent vocal fold medialisation. No patient required tracheostomy or gastrostomy, contrasting with the 25% tube-feeding dependence reported in other series [9]. Three patients received radiotherapy adjuvantly. No tumour recurrence had been identified by the last follow-up appointment.

Patient-reported outcome measures are shown in Table 2. The nerve-fibre preservation group (*n* = 3 achieved) demonstrated higher MDADI composite scores (91 versus 66) and superior UW-QOL physical and social–emotional function scores compared with the complete transection group (*n* = 3). Dysphonia was reported by five patients as mild difficulties projecting their voices in loud environments or when fatigued.

**Table 2:** Patient-reported outcome measures.

PROM		Nerve-fibre preservation	Transection	All
MDADI	Global (max 5)	5	3	4
	Physical (max 40)	39	25	30
	Emotional (max 30)	26	19	22
	Functional (max 25)	21	18	20
	Composite final (max 100)	91	66	74
UW-QOL	Physical function (max 600)	503	488	495
	Social–emotional (max 600)	503	454	478
	General QOL (max 300)	200	169	184

Abbreviations: MDADI, MD Anderson Dysphagia Inventory; UW-QOL, University of Washington Quality of Life questionnaire; PROM, patient-reported outcome measure.

## Discussion

Surgery for vagal PGL carries significant morbidity and should be carefully considered against other management options including surveillance [1]; only a selected group of patients underwent surgery at our centre. In a 20-year series of 46 vagal PGL resections, all patients developed permanent vocal fold paralysis and 25% were still dependent on tube feeding at discharge [9]. A systematic review found functional nerve preservation had been carried out in only 5% of patients [7].

Nerve-fibre preservation was achieved in 43% of the patients identified who received surgery, which is substantially higher than published rates. Preservation was only feasible when an identifiable dissection plane existed between nerve fascicles and tumour capsule. We cannot exclude that tumours amenable to preservation had more favourable characteristics, though mean tumour size did not differ substantially between groups (preservation 3.7 cm versus transection 3.9 cm). Nerve-fibre preservation did not prevent vocal fold immobility, but may have preserved some sensory and motor nerve fibres contributing to swallow function, which is consistent with the observation that in patients with pre-existing vagus nerve dysfunction, surgery causes further worsening of function [1].

The use of PROMs in vagal PGL appears to be novel. To our knowledge, MDADI and UW-QOL have not previously been reported in this population. The nerve-fibre preservation group demonstrated better long-term swallowing (MDADI composite 91 versus 66) and quality of life scores. However, these differences should be interpreted with caution given the small sample size and potential selection bias. No patient required gastrostomy, contrasting with the 25% tube-feeding rate reported elsewhere [9].

The genetics of PGLHN are increasingly understood. Pathogenic variants in *SDHD* are the most common germline variant. Tumours are rarely metastatic but frequently bilateral, with penetrance estimated to be 80%–90% by 50 years of age [10]. Pathogenic variants in *SDHD* demonstrate parent-of-origin effect, with tumour development only occurring when the pathogenic variant is inherited through the paternal line [11]. *SDHB* pathogenic variants carry a higher rate of metastatic disease [10, 12]. In the UK, 11 genes are now screened for pathogenic variants, and all patients with PGLHN should be offered genetic testing [1]. In our series, 43% had confirmed pathogenic variants in *SDH*, and 57% had multifocal disease.

The rate of metastasis in vagal PGL is reported to be <19% [13]. No patient in our series had metastatic disease, and no tumour recurrence was identified. Five-year tumour control from systematic review data is 98% after complete resections and 93% overall [7]. Our results are consistent with these published outcomes.

Limitations of the present series include the single-institution sample size precluding statistical analysis, the retrospective design, and any selection bias. The single-institution design ensures consistency in selection for surgery and surgical technique but limits external validity.

Nerve-fibre preservation was achieved in 43% of vagal PGL resections. The absence of tumour recurrence at a mean follow-up of 107 months and superior PROMs in the nerve-preservation group indicate a potential role for this technique when anatomically feasible. The natural history of PGLHN is long, and there is a paucity of prospective data to support optimal management of these rare tumours [1]. Further progress will need regionalization of head and neck paraganglioma care and prospective, multi-centre data collection.
